# The Atlantic divide: contrasting surgical robotics training in the USA, UK and Ireland

**DOI:** 10.1007/s11701-022-01399-5

**Published:** 2022-04-02

**Authors:** Tamara M. H. Gall, Gautam Malhotra, Jessie A. Elliott, John B. Conneely, Yuman Fong, Long R. Jiao

**Affiliations:** 1 Department of Surgery and Cancer, Imperial College London, Hammersmith Hospital Campus, Du Cane Road, London, UK; 2 Department of Surgery, City of Hope Medical Center, 1500 East Duarte Road, Duarte, CA 91010 USA; 3 Department of Surgery, Mater Misericordiae University Hospital, Dublin, Ireland; 4 Department of Academic Surgery, The Royal Marsden Hospital, London, UK

**Keywords:** Robotic surgery, Training, General surgery

## Abstract

The uptake of robotic surgery is rapidly increasing worldwide across surgical specialties. However, there is currently a much higher use of robotic surgery in the United States of America (USA) compared to the United Kingdom (UK) and Ireland. Reduced exposure to robotic surgery in training may lead to longer learning curves and worse patient outcomes. We aimed to identify whether any difference exists in exposure to robotic surgery during general surgical training between trainees in the USA, UK and Ireland. Over a 15-week period from September 2021, a survey was distributed through the professional networks of the research team. Participants were USA, UK or Irish trainees who were part of a formal general surgical training curriculum. 116 survey responses were received. US trainees (*n = *34) had all had robotic simulator experience, compared to only 37.93% of UK (*n = *58) and 75.00% of Irish (*n = *24) trainees (*p <  *0.00001). 91.18% of US trainees had performed 15 or more cases as the console surgeon, compared to only 3.44% of UK and 16.67% of Irish trainees (*p <  *0.00001). Fifty UK trainees (86.21%) and 22 Irish trainees (91.67%) compared to 12 US trainees (35.29%) do not think they have had adequate robotics training (*p <  *0.00001). Surgical trainees in the USA have had significantly more exposure to training in robotic surgery than their UK and Irish counterparts.

## Introduction

Robotics is the next generation of minimally invasive surgery, combining a three-dimensional visual field enhancing depth perception, with articulated instruments which provide the natural seven degrees of motion and eliminate physiological tremor. Since the year 2000, when the Da Vinci robotic system (Intuitive Surgical Inc., California, USA) gained FDA-approval [1], there has been continuous improvement in robotic technologies, and the emergence of several other robotic surgery platforms from competing companies including Versius, CMR Surgical (Cambridge, UK) and Hugo, Medtronic (Minnesota, USA).

When learning a new procedure, performance tends to improve with experience. Graphically plotting performance against experience produces a learning curve. There are four main phases: first, the commencement of training and the ascent to performance of an acceptable standard; second, the point at which the procedure can be performed competently and independently and additional experience results in small improvements in outcomes; thirdly a plateau is reached where no improvement is gained with further practice; finally, a fall in the level of performance may be seen in advancing age with reduced dexterity, eyesight and cognition [2]. Robotic surgery has a shorter phase 1 learning curve than laparoscopic surgery to acquire basic surgical skills including suturing and knot-tying [3]. Furthermore, procedural specific phase 1 learning curves are also reduced compared to laparoscopic surgery for liver resection [4], colectomy [5], rectal resection [6], nephrectomy [7] and vascular anastomoses [8]. In pancreaticoduodenectomies, a significant improvement in outcomes is seen after 40 robotic cases [9], but may be as high as 60–104 cases for laparoscopic operations [10, 11]. The use of 3D optics reduces the distraction level while operating [12], and controlling surgical instruments from a console eliminates hand dominance [13]. In addition, improved ergonomics results in increased surgeon comfort and reduced surgeon fatigue [3].

This has led to a widespread re-evaluation of conventional surgical techniques in high-income countries and a rapidly increasing availability of robotic surgery across surgical specialties. Indeed, from 2010 to 2017, there was a 2460% increase in the number of general surgical robotic operations performed in the United States of America (USA) [14]. Robotic surgery is now an important and integral component of the comprehensive care of cancer patients in the USA [15] and improved outcomes for robotic-assisted surgery compared to laparoscopy have been observed across multiple specialties [16–20], which is no surprise given that robotic surgery is laparoscopy with advanced technology.

With the initiation of new technology however, there must be adequate training to ensure that these emerging techniques are performed safely by current and future surgeons. General surgical trainees must learn this new advanced skillset to remain up to date with current trends in surgery and be able to safely deliver the robotic technique. However, the integration of robotics is not uniform across surgical training programs internationally. In 2017, 4409 Da Vinci robotic platforms were delivered globally. However, 65% of these (2862) were to the USA where 877,000 robotic operations were performed that year [14]. In England, only 25.9% of NHS Hospital Trusts have at least one surgical robot, and in 2019 only 10,067 robotic cases were performed with only 200 robotic general surgical operations [21].

The Society of American Gastrointestinal and Endoscopic Surgeons (SAGES) published a consensus document outlining desired surgical training and credentialing [22]. They state that a structured training program is required for those wishing to perform robotic surgery. Robotic privilege can be granted to those who have who have successfully completed a residency and/or fellowship program that incorporated a structured curriculum in therapeutic robotic devices and their use [22]. Exposure to robotics during surgical training should include hands-on training, including experience with the device in a dry lab environment and simulation. As well as documented experience of an appropriate volume of cases with satisfactory outcomes [22].

Inadequate robotics exposure and training is likely to lead to longer operative learning curves as an independent surgeon. This may result in poorer patient outcomes during phase 1 of the learning curve as well as reduced theatre efficiency, and a more hesitant adoption of this technique potentially resulting in large health inequalities between countries.

We aimed to identify whether there is any difference in robotics exposure, i.e. simulation and case volume as the console surgeon, between general surgery trainees in the USA, the UK and Ireland.

## Methods

Data were collected over a 15-week period from 17th September 2021. Data collection and analysis was anonymised according to General Data Protection Regulation (GDPR) guidelines. Participation was voluntary and all individuals were able to withdraw at any time prior to completion and submission of the survey. An online survey was administered and disseminated to current and recent surgical trainees from the United States of America (USA), the United Kingdom (UK) and Ireland. Participants were general surgery residents (USA) or registrars (UK and Ireland). Surgeons who were more than 12 months following the completion of training, junior surgeons below resident/registrar level, those not part of a formal general surgical training curriculum, and those training/trained in surgical specialties other than general surgery including urology and gynaecology were excluded. The survey was distributed via surgical social media platforms through the professional networks of the research team including the dissemination across Regional surgical trainee groups and National surgical trainee committee groups. The survey questionnaire consisted of 10 questions related to the participants level of training and their experience with robotic surgery during training (Appendix 1).

Statistical analysis was performed with IBM SPSS Statistics v25. Three group comparisons were made using one-way ANOVA for continuous parametric data, Kruskall–Wallis for continuous non-parametric data and chi-squared test for categorical data. Two group comparisons were made using the student *t* test, Mann Whitney *U* test and chi-squared test respectively. Results are recorded to two decimal places. A *p* value of < 0.05 was considered significant.

## Results

### Participants

Over the data collection time period, 116 survey responses were received from general surgical trainees, 34 (29.31%) from the USA; 58 (50.00%) from the UK; and 24 (20.69%) from Ireland. Participants were completing or had completed their training in eight different regions of the US (Atlanta, California, Chicago, Mid-West, Nebraska, Pacific North-West, Texas and Virginia); nine different regions of the UK (Bournemouth, London, Mersey, North–East, Oxford, Scotland, South–West, Wales and West Midlands); and four different regions of Ireland (Cork, Dublin, Galway and Leinster). Fifty-two percent of the participants had completed their surgical training and were undergoing fellowships, 36.21% were in their final two years of training and 18.97% were early year resident/registrar trainees Table 1. The majority of participants (*n = *74) chose general surgery or surgical oncology as their current specialty as well as colorectal (*n = *33); upper gastrointestinal (*n = *27); hepato-pancreato-biliary (*n = *24); breast (*n = *6) and transplant (*n = *1).

**Table 1:** Robotic experience in training of USA and UK surgical trainees

	Total (*n = *116)	USA trainees (*n = *34)	UK trainees (*n = *58)	Ireland trainees (*n = *24)	*p* value
Resident/registrar training year					0.045
1–4, *n* (%)	22 (18.97)	5 (14.71)	9 (15.52)	8 (33.33)	
5–6, *n* (%)	42 (36.21)	8 (23.53)	24 (41.38)	10 (41.67)	
Post training, *n* (%)	52 (44.83)	21 (61.76)	25 (43.10)	6 (25.00)	
Robotic simulator experience, hrs					< 0.00001*
0, *n* (%)	42 (36.21)	0 (0)	36 (62.07)	6 (25.00)	
1–10, *n* (%)	44 (37.93)	24 (70.59)	10 (17.24)	10 (41.67)	
> 10, *n* (%)	30 (25.86)	10 (29.41)	12 (20.69)	8 (33.33)	
Total number of procedures performed on robotic console					< 0.00001*
0, *n* (%)	61 (53.51)	0 (0)	47 (81.03)	14 (58.33)	
< 15, *n* (%)	18 (15.79)	3 (8.82)	9 (15.52)	6 (25.00)	
15–30, *n* (%)	19 (16.38)	16 (47.06)	1 (1.72)	2 (8.33)	
> 30, *n* (%)	18 (15.79)	15 (44.12)	1 (1.72)	2 (8.33)	
Access to robotic platform at workplace, *n* (%)	74 (63.79)	34 (100.00)	26 (44.83)	20 (83.33)	< 0.00001*
Adequate exposure to robotic surgery in training?					< 0.00001*
Yes, *n* (%)	32 (27.59)	22 (64.71)	8 (13.79)	2 (8.33)	
No, *n* (%)	84 (72.41)	12 (35.29)	50 (86.21)	22 (91.67)	

### Previous robotic training

**Fig. 1:**
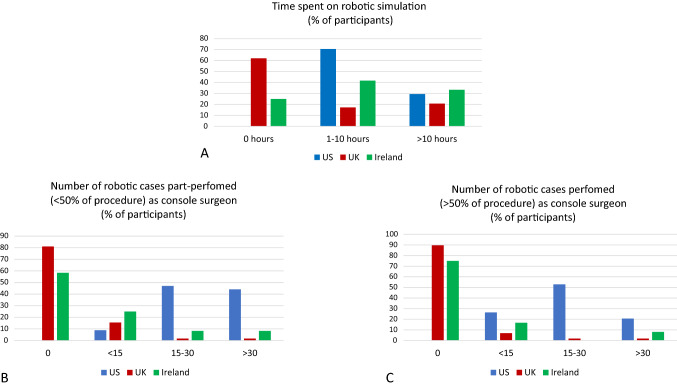
Robotic training and experience of surgical trainees from the USA and from the UK

Participants from the USA had spent significantly more time on a robotic simulator (*p <  *0.00001) and had both part-performed (less than 50% of the operation, *p <  *0.00001) and performed (more than 50% of the operation, *p <  *0.00001) more procedures on the robotic console than their UK and Irish counterparts. There was no difference between UK and Irish trainees (*p* = 0.0074; *p* = 0.11; and *p* = 0.026 respectively). Indeed, only 2 UK trainees, and 4 Irish trainees had been the console surgeon for part of the operation in at least 15 cases. Forty-seven UK trainees (81.03%) and 14 Irish trainees (58.33%) had never operated as the console surgeon for any part of an operation, compared to all USA trainees having performed more than 50% of an operation at the robotic console. Table 1, Fig. 1.

### Current status

All USA participants have access to a Da Vinci (Intuitive, California, USA) robotic platform in their current place of work, 30 (88.24%) with a dual console in the operating room. In Ireland, 20 trainees have access to a Da Vinci, 12 (50.00%) with a dual console. In the UK only 24 participants (41.38%) have access to a Da Vinci robot in their hospital with only half of these having a dual console. Two have access to a Versius robot (CMR Surgical, Cambridge, UK), while over half (*n = *32, 55.17%) have no robotic platform at their current workplace.

86.21% of UK and 91.67% of Irish trainees would like more exposure to robotic surgery compared to only 35.29% of USA trainees, the remainder stating that they have had adequate exposure to robotic training.

Of all participants, 63.79% (*n = *74) believe that their future surgical practice will involve robotic surgery in at least 25% of operations, with 31.03% (*n = *36) expecting to use a robotic technique in 50% or more of their future surgical cases. Only 6 trainees (5.26%), and all from the UK and Ireland, did not think they would perform robotic surgery in the future.

## Discussion

We surveyed 116 surgical trainees from both the USA, the UK and Ireland. Robotic surgery exposure to simulation training and operating at the console, was significantly greater for US trainees than their UK and Irish counterparts. Trainees who have early experience with robotic surgery will start their journey on the surgical learning curve. The more exposure they gain, the further along the curve they will progress. Specific robotic training curriculums which incorporate senior mentorship improve short-term patient outcomes [23].

Only 37.93% of UK and 75.00% of Irish trainees had spent time on a robotic simulator compared to 100% of US trainees. Furthermore, over 81% of UK and 58% of Irish trainees, had never operated at the robotic console. This compares to all USA trainees having performed more than 50% of an operation at the console. A robotics training program should adequately cover both basic and procedural-specific training [24]. Simulation training is the first step in basic skills training and all currently available robotic platforms have their own simulator exercises. These are user-friendly, cheap to run, efficient and reproducible [25]. Furthermore, the simulator modules are rapidly advancing and now allow procedure-specific virtual reality simulation training, with and without computer-aided guidance. Intuitive’s ion platform allows patient-specific simulation, created following 3D reconstruction of a computerised tomography (CT) scan, to enable robotic -assisted bronchoscopy, and no doubt patient-specific simulation in other procedures will follow. Robotic simulation training reduces the intra-operative surgical learning curve improving theatre efficiency and may allow some robotic naïve surgeons to perform at the same level as experts during real patient cases [26]. Procedural-specific training may involve dry lab and/or wet lab experience, replicating complex tasks, allowing surgeons to develop an understanding of the strength of the robotic instruments and to develop tissue handling -skills prior to patient operating. Complex procedures may be divided into phases and each phase will also have one or more assigned procedural videos which the trainee will watch prior to watching a proctor perform a case. This step-wise training approach enables complex operations to be performed with excellent short-term outcomes [27, 28], further improved when a specific robotics curriculum with mentorship is introduced [23]. Without adequate trainee exposure to robotic simulation and procedural operating, robotic surgery may be slower to develop with surgeons taking longer to achieve procedural competency. This results in longer operations which may frustrate the operating surgeon as well as impacting theatre efficiency and therefore ultimately hospital finances. Some consultants/attendings may be less willing to re-train in a new technique without experience as a trainee, resulting in fewer patients gaining the benefits of minimally invasive surgery.

Although responders to this survey were from varying geographical locations across the three countries, it is possible that a higher rate of US responders were from areas with strong robotic programs. Currently, robotic surgery is more common in larger hospitals with tertiary specialisation. However, in the UK and Ireland, trainees rotate between central and peripheral hospitals gaining the experience of several hospitals within their regional training programs. As such, analysing differences between the numbers of trainees based at tertiary versus secondary hospitals was not possible. Six percent of trainees listed their main specialty as breast or transplant and as such would have a limited clinical experience of robotic cases. However, over 63% listed their specialty as either general surgery or surgical oncology. On these programs in all three countries, the trainees rotate between general surgical specialties including colorectal and Upper GI/HPB.

Further, it may be the case that trainees are more likely to develop robotics training during fellowship years rather than residency/registrar training. Certainly a higher percentage (61.76%) of the US survey responders were post training fellows compared to 43.10% of UK and 25% of Irish responders. However, all US trainees bar 3, including just under 40% who were completing residency, had performed at least 15 surgical cases as the console surgeon. Only 2 UK trainees and 4 Irish trainees had performed at least 15 surgical cases as the console surgeon, despite over 40% being post training fellows. Suggesting that firstly US residents have more robotics operative case training than UK and Ireland registrars and secondly that most UK and Ireland fellowships do not include access to robotic surgery. Indeed, compared to all US trainees working in hospitals with a robotic platform, only 44.83% of UK trainees 83.33% of Irish trainees work in hospitals with access to a surgical robot. This is reflected in the 62% of UK trainees and 25% of Irish trainees who have never spent any time at a robotic simulator. Highlighting that there is no formal curriculum to gain basic robotic surgical skills during simulation in these two countries compared to the US where all survey responders had gained robotic simulation experience. US training programs are integrating robotics into their curricula as per the SAGES robotic surgery consensus document [22]. Centres must strive to be competitive in order to recruit and retain high quality trainees. In the UK and Ireland, national recruitment programs and a change of hospital every 6 months during training takes away one incentive for individual centres to develop a formal robotic training program.

Robotic surgery is increasing worldwide. Examining 169,404 procedures from The Michigan Surgical Quality Collaborative, robotic general surgery was also seen to be on the rise with an increase in case volume of 13.3% [29]. The largest increases occurring in inguinal hernia repairs (27.1% increase), anti-reflux surgery (13.8% increase) and colectomies (20.6% increase) [29]. A resultant decrease in laparoscopic surgery was observed highlighting the surgeons’ perceived benefit of a robotic technique for their minimally invasive cases. From 2013 to 2019, England saw a 410% increase in robotic surgery [21], and the Intuitive Annual Report, 2019, states an 18% annual increase in robotic procedures globally. Whilst robotics is becoming established as an efficacious and safe operative technique there are many social and economic factors that are also contributing to the uptake of robotic surgery internationally. Just under 95% of those surveyed believed that they would be performing operations with a robotic technique in their future practice. Thus, trainees are aware of the continued evolution of robotic surgery and therefore there is a necessity to pay close attention to education and training. The introduction of formal robotics training is likely to further motivate trainees who will be determined to develop their skills whilst under close supervision. Skills they believe are needed for their future independent practise.

This survey was only a snapshot of trainees in each of these countries. The authors acknowledge that due to distribution over multiple networking platforms we are not able to give the true participant reposnse rate. We suspect that those more likely to complete the survey may be robotic enthusiasts. However, we feel that the discrepancy between training in the USA and in the UK and Ireland has been highlighted.

Barriers to robotic training and exposure in the UK and Ireland may be due to cost, access and training. We believe that with the emergence of robotic competitors the cost will decline. Further, as more hospitals buy or rent more robotic platforms, the individual operative cost is likely to significantly reduce as seen with a thirty-year history of laparoscopy. Access to a robotic theatre will therefore also improve as robotics becomes more and more standardised in some centres. A surgeon should have access to a robotic theatre and/or simulation at least once a week for continued professional development and maintenance of technical skills. With heavy investment into robotics in the UK and Ireland we would hope this to be the case in the majority of hospitals in the future. We expect the UK and Ireland to follow the USA over the next decade, with more surgical trainees exposed to robotic surgery and training as standard. However, developing UK and Ireland robotic training centres and implementing robotics into national training programs, before current more senior surgeons are through their initial learning curve, will significantly reduce this time-lag and ensure a safer execution of robotic surgery for patients.

Although robotic surgery will not replace other surgical techniques completely, there is a definite increase in the number of cases performed and we expect this trend to continue. Surgical trainees must be exposed to this technique to develop their technical skills and minimally invasive procedural knowledge. Training and experience improves surgical skill, thus improving patient outcomes. Ultimately, a lack of exposure to robotics during training in the UK and Ireland compared to the US may lead to a significant variation in future surgical practice and short-term patient outcomes between these high-income countries.

This survey has called attention to the growing divergence between USA and UK and Ireland surgical trainees’ access to robotics simulation and procedural training in general surgery. This emphasises the necessity to introduce standardised robotics training into the general surgery curriculum.

## Appendix 1 Robotic surgery training survey

**Table:**

1. In which country are you completing your surgical training?	
USA	
UK	
Ireland	
2. In which region/ hospital have you completed most of your training?	
3. What year of your residency/registrar training are you?	
1	
2	
3	
4	
5	
6	
Post residency/CCT Fellow	
Post fellowship	
4. What is your current specialty/sub-specialty? Tick all that apply	
General Surgery	
Surgical Oncology	
Colorectal	
UGI	
HPB	
Breast	
Endocrine	
Vascular	
Other (please specify)	
5. How many hours in total have you spent on a robotic simulator (simulator, dry lab, wet lab)	
0	
1–10	
More than 10	
6. How many operations have you part-performed on the robotic console (< 50% of the operation)?	
0	
< 15	
15–30	
More than 30	
7. How many operations have you performed on the robotic console (> 50% of the operation)?	
0	
< 15	
15–30	
More than 30	
8. Does your current place of work have at least one Da Vinci robot?	
Yes but no dual console	
Yes with dual console	
No but other robotic platform in use (CMR/Medtronic/other)	
No	
9. Do you think you have had enough exposure to robotic surgery?	
Yes	
No, would like more	
No, not necessary to have more	
10. Do you think your future practice over the next 10 years will include robotic surgery?	
Yes but < 25%	
Yes 25–50%	
Yes > 50%	
No	
